# Functional foods and exercise in breast cancer: epigenetic modulation, chemotherapy tolerance, and fatigue management

**DOI:** 10.3389/fnut.2025.1622597

**Published:** 2025-08-13

**Authors:** Baolei Ma, Haidong Liu

**Affiliations:** ^1^Sports Department of Xi'an Polytechnic University, Xi'an, Shaanxi, China; ^2^School of Physical Education, Chengdu Normal University, Chengdu, Sichuan, China

**Keywords:** exercise, functional foods, breast cancer, performance, epigenetic modulations

## Abstract

**Background:**

Breast cancer (BC) is the most commonly diagnosed malignancy among women worldwide. There is increasing interest in the role of modifiable lifestyle factors, particularly nutrition and physical activity, in influencing cancer risk, progression, and treatment response.

**Objective:**

This review explores how functional foods and exercise can modulate BC through molecular and epigenetic mechanisms and evaluates their potential as adjunctive strategies in prevention and therapy.

**Findings:**

Functional foods, such as those rich in polyphenols, omega-3 fatty acids, vitamins, and probiotics, impact BC biology through DNA methylation, histone modification, and microRNA regulation. Exercise similarly modulates key pathways related to inflammation, immune function, hormone balance, and apoptosis. Combined interventions show synergistic potential in reducing tumor growth, enhancing therapy response, and improving quality of life.

**Conclusion:**

Functional foods and exercise represent promising, non-toxic strategies for modulating BC risk and progression via epigenetic and cellular pathways. However, more clinical trials are needed to define optimal combinations and dosages. Future research should focus on precision-based, lifestyle-integrated cancer care approaches.

## 1 Introduction

Food serves three primary purposes: (1) it provides energy through carbohydrates, proteins, and fats, along with essential nutrients; (2) it offers enjoyment through appealing aromas, colors, and flavors; and (3) it can confer health benefits (1, 2). Functional foods resemble conventional foods and are typically included in a regular diet, but they also provide physiological advantages and may lower the risk of chronic diseases beyond basic nutritional value. Functional foods influence biological processes in the body, offering health benefits in several key areas of human physiology (2). Certain functional foods contain bioactive compounds that may help reduce the risk of cancer by inhibiting tumor growth, enhancing immune function, and promoting apoptosis in cancerous cells (3).

**Figure 1:**
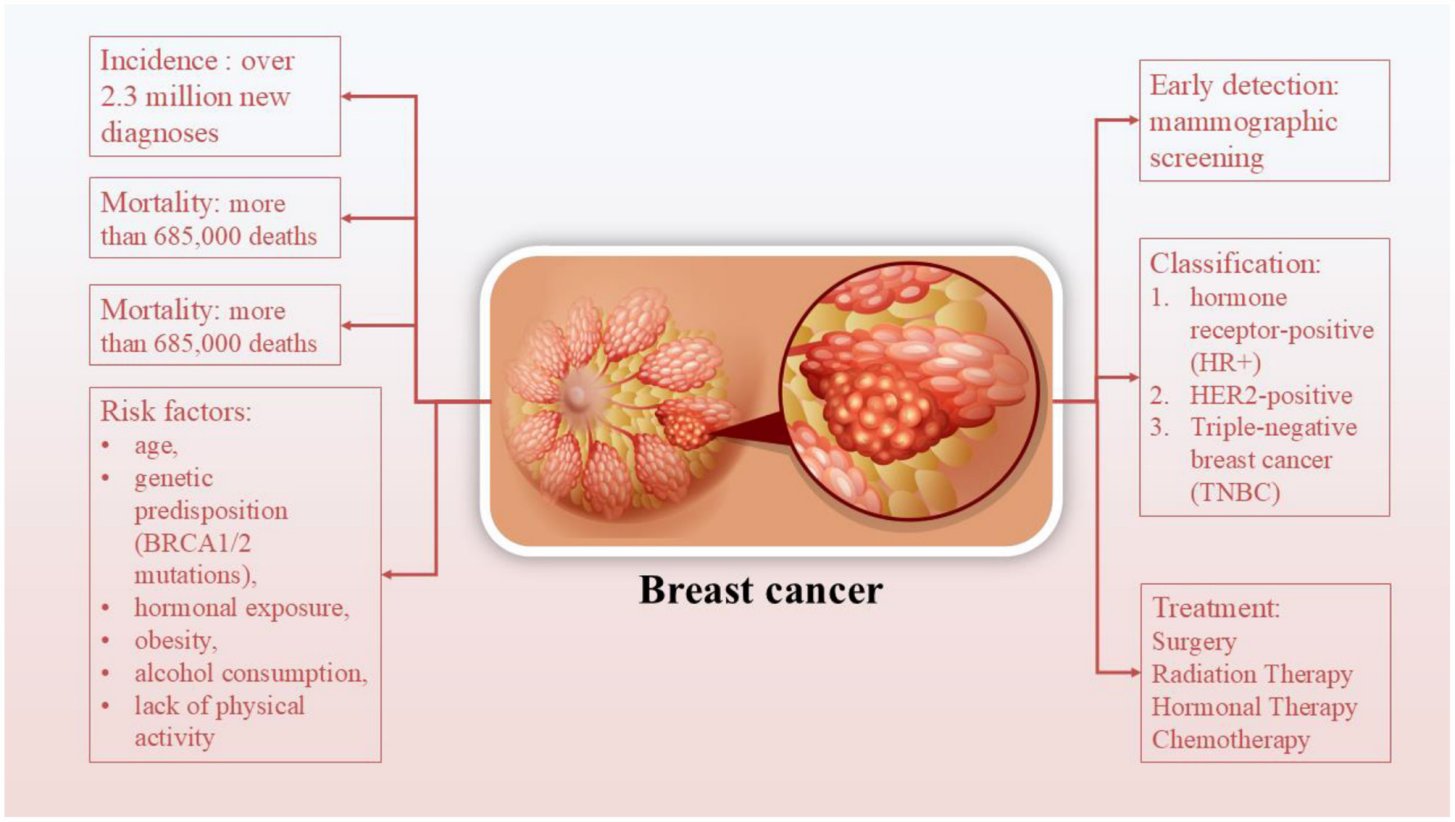
Breast cancer and its incidence, mortality, risk factors, and treatments.

Breast cancer (BC), in particular, stands out as one of the most frequently diagnosed cancers worldwide and remains a leading cause of cancer-related mortality among women (4, 5) (Figure 1). It accounts for ~11.7% of all new cancer cases globally, with over 2.3 million new diagnoses and more than 685,000 deaths reported in 2020, according to the World Health Organization. The incidence continues to rise, particularly in low- and middle-income countries, due to increasing life expectancy, urbanization, lifestyle changes, and limited access to screening programs (6, 7). Early detection through mammographic screening and public awareness has improved survival rates, particularly in high-income nations, yet disparities in outcomes persist across regions (6). BC encompasses a heterogeneous group of diseases classified based on molecular characteristics into subtypes, such as hormone receptor-positive (HR+), HER2-positive, and triple-negative BC (TNBC) (6, 7). Treatment strategies for BC are multimodal and depend on the tumor subtype, stage, and individual patient factors. They typically include a combination of surgery, radiation therapy, chemotherapy, and hormonal therapy (8). It seems that patients who undergo surgery may require a substantial amount of time before recovering their Health-Related Quality of Life (HRQoL). Similarly, chemotherapy and radiation may also cause physical and physiological unwanted effects for patients, which frequently include fatigue, insomnia, nausea and vomiting, neuropathy, cardiotoxicity, and strength impairment (9). Due to BC complexity and its multiple side effects, life style-related approaches are required for reducing the unwanted side effects. Among complementary approaches, exercise is particularly effective in either preventing BC or mitigating decrements in indices of physical fitness. The results of a meta-analysis show that exercise interventions for BC patients, all of which were part of adjuvant treatment, effectively reduced declines in fitness and the worsening of symptoms observed in control groups (CGs). Notably, following radiation therapy (RT) protocols, there were small increases in fatigue symptoms (+5.95%), which were more pronounced in all other intervention protocols, along with improvements in other measures [by +6.4%, +21.9%, and +12.1% for cancer-related fatigue (CRF), strength (ST), and HRQoL, respectively]. However, the specific impact of combined (COMB) protocols in BC patients remains unclear, as only a single study involving COMB intervention was included (9). The findings related to breast cancer survivors (BCS) showed enhancements in physical fitness indicators following exercise interventions, whereas no changes were noted in the control groups. Both the COMB and RT interventions for BCS displayed promising results, with decreases in fatigue and improvements in cardiorespiratory fitness, strength, and health-related quality of life. Notably, RT interventions resulted in greater percentage increases in health-related quality of life and strength, along with a more significant decrease in fatigue compared to the changes noted for aerobic interventions. Furthermore, positive outcomes were seen with Pilates and Yoga interventions, although additional studies are needed to confirm these results (9).

Another meta-analysis in 2022 reviewed 20 randomized clinical trials involving 1,793 participants and found that physical exercise is effective in reducing fatigue in BC patients (10). They also declared that the supervised combination of resistance training (RT) with aerobic training (AT) is the most effective protocol for reducing fatigue, especially for women undergoing chemotherapy (10). Other than fatigue, trials reported significant improvement in muscle strength, pain, and minor changes in aerobic capacity. These results are also confirmed by previous systematic reviews (11, 12).

Functional foods and their bioactive compounds have shown promise in influencing various biological pathways, including those involved in epigenetic regulation, such as DNA methylation, histone modification, and non-coding RNA expression, that play critical roles in cancer initiation and progression (13). In light of this, our review aims to critically examine current evidence on how functional foods modulate epigenetic processes in BC, with particular focus on DNA methylation, histone modification, and non-coding RNA regulation. Furthermore, we explore how these epigenetic changes interact with exercise-based interventions to influence treatment outcomes, tumor suppression, and survivorship, thereby highlighting their potential as preventive or adjunctive therapeutic strategies. To the best of our knowledge, this is one of the first reviews to comprehensively synthesize data on the combined impact of functional foods and exercise on epigenetic modulation and programmed cell death in BC, highlighting their potential for use in precision lifestyle medicine.

## 2 Epigenetics: what do we know?

### 2.1 DNA methylation

In animals, the methylation of DNA occurs on the cytosine nucleotide and predominantly at CpG dinucleotides. Interestingly, these CpG dinucleotides are found in the human genome at approximately one-fifth of their expected frequency (14). Additionally, they tend to cluster together, forming regions known as “CpG islands.” These CpG islands are essential for regulating gene expression. The sequencing of the human genome has shown that nearly half of all human genes contain CpG islands within their regulatory regions. This suggests that DNA methylation at these CpG islands may be a key mechanism for achieving cell type-specific gene expression during development. Generally, the silencing of gene expression through epigenetic mechanisms is linked to the hypermethylation of CpG islands (14). In BC tissues, specific tumor suppressor genes often exhibit hypermethylation, which can be identified in the early stages of the disease. The alterations in DNA methylation observed between healthy and cancerous breast tissues may serve as valuable prognostic and diagnostic biomarkers for BC (15). Avraham et al. (16) investigated differences in DNA methylation patterns between healthy breast tissue, other normal tissues, and cancerous tissues from the breast, colon, lung, and endometrium. Their findings revealed that several genes exhibited distinct methylation profiles in malignant breast tissue (16). In non-cancerous tissues, the promoters of most of these genes showed low levels of methylation, whereas in cancerous tissues, including breast tumors, the same gene promoters were hypermethylated and associated with reduced gene expression. Interestingly, TRIM29 displayed a unique pattern: it was hypomethylated in normal breast tissue but hypermethylated in other healthy tissues. In contrast, in breast tumors, TRIM29 showed promoter hypermethylation and decreased expression, while in other tumor types, its promoter was hypomethylated. These findings suggest that specific epigenetic changes may contribute to tissue-dependent cancer risk and tumor development (16).

In BC tissues, specific tumor suppressor genes often exhibit hypermethylation, which can be identified in the early stages of the disease. The alterations in DNA methylation observed between healthy and cancerous breast tissues may serve as valuable prognostic and diagnostic biomarkers for BC (15). Numerous gene-specific changes in DNA methylation have been discovered, indicating that these epigenetic modifications could hold prognostic significance in BC (17). One notable example is E-cadherin, a protein that plays a crucial role in cell-cell adhesion through its interaction with catenin. When the E-cadherin gene is silenced due to genetic or epigenetic modifications, it can contribute to tumor development. A study mapped the methylation patterns of the E-cadherin gene promoter in 50 BC tissues and compared them to 50 normal breast samples. Consistent with earlier research, the findings revealed that 94% of the cancerous tissues exhibited hypermethylation of the E-cadherin promoter, which was linked to a more aggressive tumor phenotype in infiltrating BC (17). Avraham et al. (16) investigated differences in DNA methylation patterns between healthy breast tissue, other normal tissues, and cancerous tissues from the breast, colon, lung, and endometrium. Their findings revealed that several genes, such as ALX4, FEV, HOXA11, LYL1, NEUROG1, PAX9, MGMT, SOX10, SREBF1, TP73, and TRIM29, exhibited distinct methylation profiles in malignant breast tissue (16). In non-cancerous tissues, the promoters of most of these genes showed low levels of methylation (hypomethylation), whereas in cancerous tissues, including breast tumors, the same gene promoters were hypermethylated and associated with reduced gene expression. Interestingly, TRIM29 displayed a unique pattern: it was hypomethylated in normal breast tissue but hypermethylated in other healthy tissues. In contrast, in breast tumors, TRIM29 showed promoter hypermethylation and decreased expression, while in other tumor types, its promoter was hypomethylated. These findings suggest that specific epigenetic changes may contribute to tissue-dependent cancer risk and tumor development (16). De Almeida et al. (18) conducted a comparative analysis of DNA methylation and gene expression in BC tissues and their corresponding normal counterparts. Their study identified methylation not only in genes previously linked to cancer, such as WT1, ZNF154, BCL9, HOXD9, SMYD3, ITIH5, and ZNF177, but also in seven additional genes, RNF220, TMEM132C, TDRD10, EFCAB1, RIMBP2, ANKRD53, and PRAC2 (also known as C17orf93), which had not been previously associated with oncogenic processes (18). These newly implicated genes showed elevated promoter methylation levels in tumor tissues compared to normal tissues, which was generally accompanied by reduced gene expression. When assessing the prognostic significance of CpG site methylation, it was found that only PRAC2, TDRD10, and TMEM132C had meaningful correlations with patient survival outcomes. Interestingly, in tumor samples, PRAC2 expression was increased, while TMEM132C and TDRD10 showed reduced expression levels, despite all three exhibiting promoter hypermethylation. These findings suggest that these genes may play a role in tumor biology and could serve as potential prognostic biomarkers in BC (18). Taken together, evidence shows that DNA methylation changes are common in BC and often involve hypermethylation of tumor suppressor genes, which can be detected early and may serve as useful diagnostic and prognostic biomarkers.

### 2.2 Histone modification

Genetic material in the nucleus is organized into compact structures called nucleosomes, which consist of ~146 base pairs of DNA wrapped around an octamer of histone proteins—two each of H2A, H2B, H3, and H4. The histone proteins possess flexible N- and C-terminal tails that extend outward from the nucleosome core. These “histone tails” are the primary sites for a variety of post-translational modifications (PTMs), which modulate the interactions among nucleosomes and between DNA and histones, thereby influencing chromatin structure and gene activity (19). Two of the most extensively studied modifications are acetylation and methylation. Acetylation, catalyzed by histone acetyltransferases (HATs), typically targets lysine residues in histones H3 and H4 using acetyl-CoA, and is associated with gene activation due to loosening of chromatin structure. Conversely, histone deacetylases (HDACs) remove acetyl groups, often resulting in transcriptional repression and gene silencing. Methylation of histones is mediated by histone methyltransferases (HMTs), which use S-adenosylmethionine (SAM) as a methyl donor to target lysine or arginine residues on histone tails (20).

Research indicates that histone modifications (HMs) are crucial in the tumorigenesis and progression of BC, with alterations in global patterns of these modifications leading to varying effects on the disease. In a study involving 880 BC specimens, seven histone modification markers (H3K9ac, H3K18ac, H4K12ac, H4K16ac, H3K4me2, H4K20me3, and H4R3me2) were analyzed, revealing a negative correlation between the expression of all seven markers and tumor grade (21). Additionally, elevated levels of H4R3me2 and H3K9ac were found in specimens with lower lymph node involvement, while H4R3me2, H3K9ac, and H4K16ac levels were reduced in larger tumors (21). Notably, low or absent H4K16ac was observed in the majority of samples, suggesting that this alteration may represent an early event in BC development and could potentially serve as a diagnostic marker. Furthermore, higher levels of these histone markers in various BC subtypes were linked to a more favorable prognosis, predominantly found in luminal BC. In contrast, lower levels of these markers were noted in triple-negative breast cancer (TNBC) and HER2-positive BC, both of which are associated with poorer outcomes (21). During the malignant transformation of breast tissue, there is a noticeable global alteration in HMs (22). For example, a study of 58 breast samples found that levels of acetylated histone H4, H4K12ac, and acetylated tubulin, as well as the histone deacetylases HDAC1, HDAC2, and HDAC6, were significantly lower in ductal carcinoma *in situ* (DCIS) and invasive ductal carcinoma (IDC) compared to normal mammary epithelium (22).

In response to DNA double-strand breaks, activated members of the PI3K family, along with ataxia-telangiectasia mutated (ATM) and ATM- and Rad3-Related (ATR), facilitate the phosphorylation of histone H2AX, resulting in the formation of γH2AX. This modified form of H2AX serves as a biomarker for DNA damage and repair, initiating cell cycle checkpoints and the repair of double-strand breaks. In BC, γH2AX is linked to reduced expression of estrogen receptors (ERs) and progesterone receptors (PRs), as well as unfavorable clinicopathological features. These include larger tumor size, higher tumor grade, and increased lymph node involvement (23, 24). Moreover, various HMs play a crucial role in either activating oncogenes or inhibiting tumor suppressor genes (TSGs). This can result in sustained proliferative signaling, accelerated cell cycles, enhanced angiogenesis, invasion, and metastasis, DNA damage, resistance to apoptosis, reprogramming of energy metabolism, and evasion of immune destruction. Overall, HMs have emerged as important biomarkers for the diagnosis and prognosis of BC. Investigating the mechanisms behind these modifications also offers potential for the development of targeted inhibitors. The specific histone modifications and their unique functions in BC will be explored in the subsequent sections. Histone modifications (HMs) play a significant role in the development and progression of BC. Studies have shown that global changes in HM patterns are associated with tumor grade, size, lymph node involvement, and clinical outcomes.

### 2.3 Non-coding RNAs

Long non-coding RNAs (lncRNAs) have emerged as vital regulators of gene expression, significantly influencing the hallmarks of cancer. By engaging in diverse mechanisms, such as chromatin remodeling, transcriptional regulation, and modulation of post-transcriptional processes, lncRNAs contribute to the dynamic regulatory landscape of the cell (25). LncRNAs are transcripts longer than 200 nucleotides that do not encode proteins. They function through mechanisms, such as guiding chromatin modifiers, acting as scaffolds for protein complexes, serving as decoys for transcription factors or microRNAs (miRNAs), and modulating mRNA splicing and stability. The dysregulation of lncRNAs has been linked to a variety of cancers, influencing cellular proliferation, apoptosis, angiogenesis, and resistance to treatments (26).

Since the initial report of miRNA deregulation in BC in 2005 (27), numerous studies have been conducted to examine the expression of various miRNAs and their functions in the context of BC. A recent systematic review (2018) in this field has reported that among all the studied miRNAs, mir-21 and miR-210 are upregulated in BC cells while miR-145, miR-139-5p, miR-195, miR-99a, miR-497 and miR-205 are reduced; however, these miRNAs need validation in BC (28). The most recent study in 2025 has worked on the expression levels of miR-222 in HER2-negative BC patients undergoing anthracycline-based chemotherapy (29). miR-222 was identified as highly upregulated in adriamycin-resistant BC cells, with PTEN confirmed as its direct target and FN1 as a key downstream effector. Engineered extracellular vesicles (EVs) carrying a miR-222 inhibitor were developed to reverse drug resistance by restoring PTEN/FN1 signaling. Molecular docking confirmed stable PTEN-FN1 interactions, and *in vivo* experiments showed that EV-delivered miR-222 inhibitors suppressed tumor growth in resistant xenograft models. These findings reveal a novel EV-mediated mechanism of drug resistance via the miR-222/PTEN/FN1 axis and suggest engineered EVs as a potential therapy, with serum EVs offering a non-invasive biomarker for treatment monitoring (29).

Another recent study investigated the role of miR-770-5p as a post-transcriptional regulator of XBP1. Using bioinformatics analyses of BC datasets, XBP1 was found to be overexpressed in tumors and associated with poorer clinical outcomes. Expression analyses, including qRT-PCR, demonstrated a significant inverse correlation between miR-770-5p and XBP1, especially in Luminal A BC cases with wild-type p53 (30). Mechanistically, miR-770-5p was shown to suppress the spliced, active form of XBP1 (XBP1s), thereby attenuating its transcriptional activity. Importantly, this suppression also influenced ESR1 (estrogen receptor alpha) expression, revealing a link between UPR modulation and hormonal signaling. Functional assays indicated that overexpression of miR-770-5p reduced ESR1 levels and enhanced sensitivity to tamoxifen, suggesting that this microRNA may reverse endocrine resistance by targeting the XBP1/ESR1 axis (30).

In conclusion, miR-770-5p exerts a tumor-suppressive role in luminal BC by targeting XBP1, downregulating ER signaling, and increasing tamoxifen responsiveness. These findings highlight the therapeutic potential of miR-770-5p as a novel strategy to overcome endocrine resistance by interfering with the IRE1α/XBP1-mediated UPR pathway (30). miR-10b has also been strongly linked with aggressive features in primary breast tumors, including larger tumor size, higher histological grade, increased vascularization, and HER2 positivity (31). Conversely, its expression tends to be lower in estrogen and progesterone receptor–positive cancers (31). These variations, both across and within tumor subtypes, contribute to inconsistent results when comparing miR-10b levels in tumors vs. normal tissue. Despite this variability, miR-10b has shown consistent correlations with poor prognosis indicators, prompting interest in its potential use as a diagnostic and prognostic biomarker (32). It is detectable in circulating tumor cells and extracellular vesicles, making it suitable for non-invasive serum-based testing. Studies report that serum miR-10b levels can distinguish BC patients from healthy individuals with high sensitivity and specificity, surpassing traditional markers such as CA15-3 and CEA (33). It has also been associated with relapse risk and poor overall survival, and levels tend to decline after surgery and radiotherapy, suggesting utility in monitoring treatment response. Emerging research also proposes its use in predicting therapy response and chemotherapy-induced anemia (34). Most notably, miR-10b appears consistently upregulated in metastatic BC, including cases with lymph node involvement (35). One study showed a 4.4-fold increase in serum miR-10b in node-positive patients vs. node-negative, with solid diagnostic potential. This upregulation is evident at various stages of metastasis, including in metastatic lymph nodes compared to matched primary tumors, further linking miR-10b with BC dissemination, especially in HER2-positive and hormone receptor–negative subtypes (35). Taken together, a diversity of miRNAs is dysregulated in BC cells which not only take part in pathophysiology of BC but also can be used as prognostic and diagnostic biomarkers, as well as therapeutic targets.

## 3 Exercise and its role in BC

Physical activity has emerged as a powerful adjunct therapy in the management of BC, influencing risk, progression, recurrence, and survivorship. Beyond its established benefits for general health, exercise can induce profound hormonal, metabolic, and immune changes that may directly impact tumor biology and patient outcomes (36, 37).

### 3.1 Types of exercise: aerobic, resistance, and high-intensity interval training (HIIT)

Three main forms of exercise, aerobic training, resistance training, and HIIT, have been studied for their effects in BC populations. Aerobic exercise, such as walking, running, or cycling, improves cardiovascular fitness and promotes systemic anti-inflammatory effects. Resistance training, involving weight lifting or bodyweight exercises, is particularly beneficial for maintaining muscle mass and combating cancer-related fatigue and sarcopenia, especially in patients undergoing chemotherapy or hormonal therapy. HIIT, which alternates short bursts of intense activity with periods of rest, has gained attention for its time efficiency and potential to improve both metabolic health and physical fitness, even in clinical populations. These exercise modalities can be used independently or combined in a tailored regimen to suit patient preferences, treatment phases, and overall health status (37).

### 3.2 Hormonal, metabolic, and immune effects

Exercise influences multiple biological systems that are relevant to BC pathophysiology:

I. Hormonal regulation: Physical activity can lower circulating estrogen levels by reducing adipose tissue, a primary site of estrogen production post-menopause. It may also improve insulin sensitivity and reduce insulin-like growth factor 1 (IGF-1), which has been linked to cancer progression.

II. Metabolic effects: Exercise enhances mitochondrial function, increases lipid oxidation, and improves glucose metabolism. These changes may contribute to a less favorable environment for tumor growth.

III. Immune modulation: Regular exercise has been shown to enhance immune surveillance by increasing the activity of natural killer (NK) cells, cytotoxic T cells, and macrophages, potentially improving the body's ability to detect and destroy malignant cells (37).

### 3.3 Synergistic potential with diet

The combination of exercise and dietary interventions can exert synergistic effects in BC care. Together, they can more effectively modulate inflammatory markers, optimize body composition, and regulate key metabolic pathways. Diets rich in antioxidants, fiber, and phytoestrogens may complement the beneficial effects of exercise on insulin sensitivity and inflammation. Moreover, integrating lifestyle interventions can empower patients with a sense of control over their health, enhancing psychological wellbeing and treatment compliance. As such, exercise is increasingly viewed not only as a supportive therapy but as a cornerstone of holistic BC management (38).

## 4 Functional foods affect BC through epigenetics

### 4.1 Curcumin

**Figure 2:**
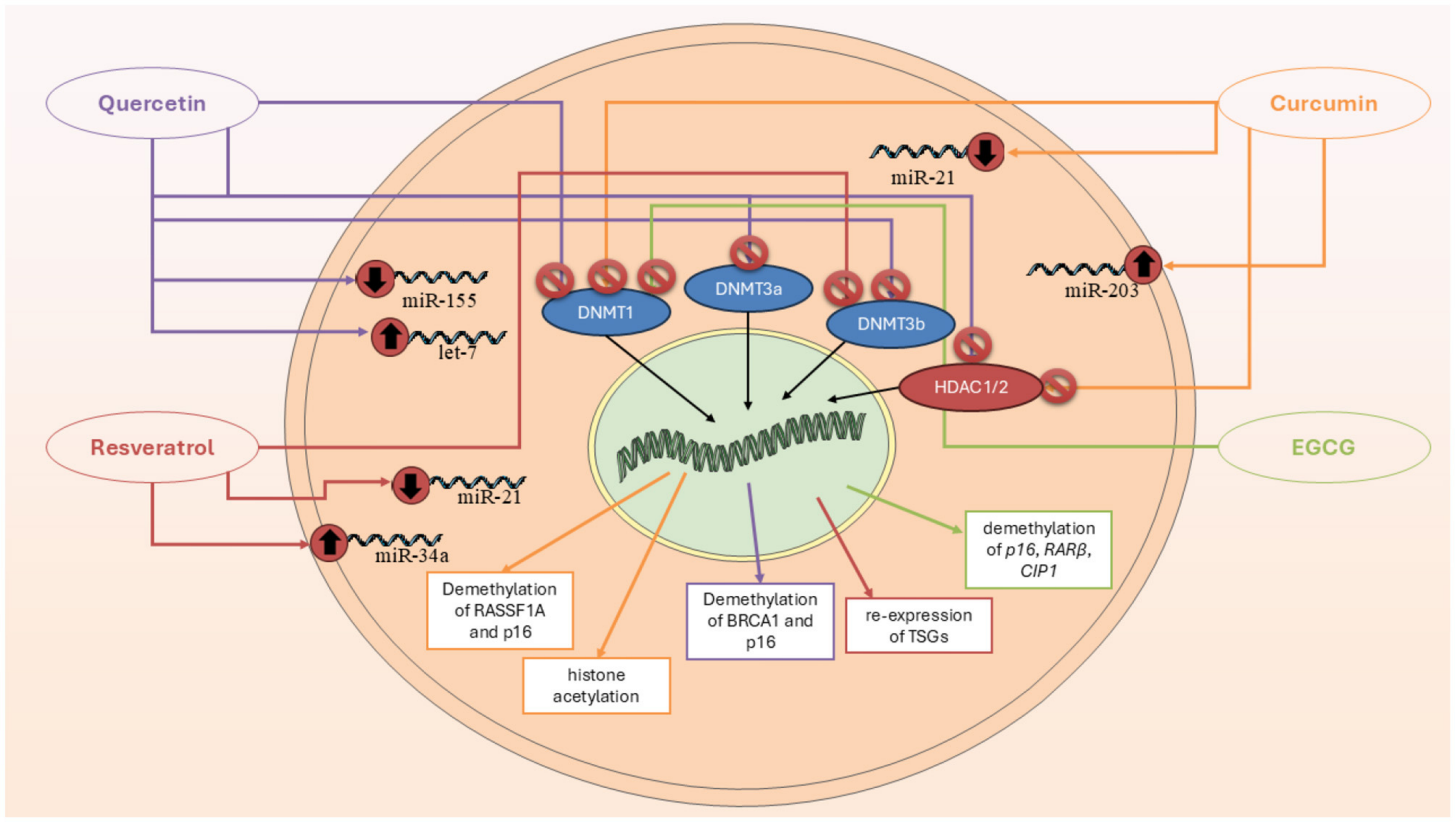
Epigenetic mechanisms through which key polyphenols—curcumin, quercetin, resveratrol, and EGCG—modulate breast cancer progression. These compounds regulate DNA methylation, histone modification, and microRNA expression, resulting in tumor suppressor gene reactivation, cell cycle arrest, and apoptosis.

Curcumin is a polyphenolic molecule known for its distinctive yellow color and wide array of biological activities (39). Curcumin's structure allows it to engage in various molecular interactions, influencing a variety of biochemical pathways and cellular processes (39). The structure of curcumin also makes it highly reactive. This reactivity contributes to curcumin's ability to modulate gene expression and epigenetic modifications, making it an attractive molecule for therapeutic applications (40). There are plenty of studies which tried to approve the epigenetic effects of curcumin (CUR) on BC cells (Figure 2). The most recent study in 2024 aimed to investigate the molecular and epigenetic effects of curcumin on MCF-7R BC cells, a multidrug-resistant cell line derived from MCF-7 cells, particularly focusing on its ability to reverse drug resistance mediated by P-glycoprotein (P-gp) and related gene regulation pathways. They observed that CUR modulates the expression of genes associated with DNA methylation and transcriptional regulation, including ABCB1/MDR1. Through reduced representation bisulfite sequencing, curcumin was shown to induce significant epigenetic alterations. These changes affected key cellular functions, such as ribosome biogenesis and cytoskeletal organization. Importantly, curcumin treatment partially reversed the drug-resistant phenotype of MCF-7R cells, making them more similar to drug-sensitive parental cells. This suggests curcumin's potential as an adjuvant therapy for overcoming chemoresistance via epigenetic reprogramming (41). Another study aimed to evaluate the expression of miR-155-5p in peripheral white blood cells (WBCs) of BC female carriers of BRCA1 methylation (42). They found out that miR-155-5p was significantly upregulated in BRCA1-hypermethylated BC cell lines compared to BRCA1-mutated (HCC-1937) and wild-type BRCA1 (MDA-MB-321) lines. Curcumin treatment suppressed miR-155-5p expression in HCC-38 cells by restoring BRCA1 expression, but had no effect in BRCA1-mutated HCC-1937 cells. Clinically, elevated miR-155-5p levels were observed in WBCs from patients with non-aggressive, localized BC, late-stage aggressive OC, and CF BRCA1-methylation carriers. IL2RG expression was decreased in OC patients and CF carriers but not in BC patients. These findings suggest that miR-155-5p plays a context-dependent role and may serve as a potential biomarker for cancer risk in BRCA1-methylation carriers (42).

Another study also worked on DNA methylation in BC cells and found that two derivatives of CUR, named ST08 and ST09, induced widespread epigenetic changes (43). WGBS revealed differentially methylated sites (DMSs) enriched in promoter regions, with 12 commonly hypomethylated genes, 50% of which are known to be methylated in patient samples and belong to the homeobox transcription factor family. Gene body methylation analysis showed hypermethylation of 910 and 952 genes in ST08- and ST09-treated cells, respectively. Notably, CACNAH1 and CDH23 were upregulated in response to ST08 and ST09, respectively. Integrated WGBS and RNA-seq analysis revealed drug-specific pathway alterations: ST08 primarily affected the extracellular matrix (ECM) pathway, while ST09 targeted the cell cycle pathway, highlighting distinct epigenetic and transcriptomic signatures for each derivative (43). Al-Yousef and colleagues (44) investigated whether curcumin can modulate DNA promoter methylation to restore normal expression of cancer-related genes—specifically reactivating the hypermethylated BRCA1 tumor suppressor and suppressing the hypomethylated SNCG proto-oncogene—in BC cell lines, including TNBC. Curcumin treatment (5 and 10 μM for 6 days) reactivated BRCA1 expression in TNBC (HCC-38) and ER-/PR- (UACC-3199) cells by reducing BRCA1 promoter methylation. It also suppressed SNCG expression in ER+/PR+ (T47D) cells by inducing promoter hypermethylation. Unlike curcumin, the DNA demethylating agent 5′-aza-2′-deoxycytidine restored BRCA1 expression only in UACC-3199, not in HCC-38 cells (44).

Another study aimed to evaluate the effect of curcumin on the expression of PRMT5 and its cofactor MEP50—key epigenetic regulators implicated in cancer progression (45). Curcumin treatment (2 and 20 μM) significantly downregulated the expression of both PRMT5 and MEP50. It also reduced levels of symmetrical dimethylarginine (SDMA) and the H4R3me2s histone mark, indicating a decrease in PRMT5 enzymatic activity. The suppression of PRMT5 expression was associated with reduced levels and promoter binding of transcription factors Sp1 and NF-YA, which bind GC-rich regions of the PRMT5 promoter. Curcumin-mediated downregulation of Sp1 and NF-YA was linked to interference in the PKC-p38-ERK-cFos and AKT-mTOR signaling pathways. These findings reveal PRMT5-MEP50 as a novel molecular target of curcumin, suggesting its potential in epigenetic-based cancer therapies (45). Kumar et al. (46) aimed to investigate whether curcumin can reverse the epigenetic silencing of the glutathione S-transferase pi 1 (GSTP1) tumor suppressor gene by modulating promoter methylation in MCF-7 BC cells. Curcumin treatment reversed hypermethylation of the GSTP1 promoter in a dose-dependent manner and reactivated GSTP1 protein expression. Specifically, 10 μM curcumin, a non-toxic dose, was sufficient to completely demethylate the GSTP1 promoter and restore gene expression after 72 h, whereas concentrations below 3 μM had no effect. The IC50 value of curcumin in MCF-7 cells was found to be 20 μM, indicating that the effective hypomethylating dose was well below cytotoxic levels. These findings support curcumin as a promising and safe hypomethylating agent capable of restoring tumor suppressor gene function in BC (46).

### 4.2 Resveratrol (RSV)

Similar to curcumin, RSV is also an important polyphenolic compound with several effects, including epigenetic regulator (Figure 2) (47). The most recent study in this field was conducted in 2024, which explores the role of Methyl-CpG binding domain (MBD) protein**s** in regulating the expression of BRCA1, BRCA2, and p16 genes, and the potential molecular mechanism through which RSV influences these processes in BC cells (47). RSV showed an IC50 value of 30 μM and significantly influenced the expression of MBD proteins, particularly MBD2, which interacted with the BRCA1 gene promoter. Treatment with higher concentrations of RSV reduced colony and sphere formation, decreased cell migration, and increased the number of apoptotic cells in ER+, PR+, and triple-negative BC cells. The MBD2-BRCA1 axis was identified as crucial in promoting apoptosis and reducing proliferation and metastasis in BC cells (47). Another study highlights how RSV effects by epigenetically silencing key regulators of oncogenic signaling in BC. Using ER-positive MCF-7 and triple-negative MCF10CA1a BC cell lines, researchers showed that a 9-day treatment with RSV (15 μM) increased DNA methylation at the enhancers of GLI2 and WNT4, critical upstream regulators of the Hedgehog and Wnt pathways (48). A phospho-protein array further confirmed that RSV inhibited Wnt signaling by downregulating activators and upregulating inhibitors of the pathway (48). In MCF-7 and MDA-MB-231 cells treated with 20 μM RVT for 48 h, there was a marked upregulation of BRCA1, p53, and p21, accompanied by a downregulation of the histone methyltransferases PRMT5 and EZH2. This led to reduced levels of their respective repressive histone marks, H4R3me2s and H3K27me3. Additionally, RSV decreased lysine deacetylase (KDAC1-3) expression and overall deacetylase activity while increasing levels of the acetyltransferases KAT2A and KAT3B, resulting in higher global levels of activating histone acetylation marks H3K9ac and H3K27ac. These findings suggest that RSV reactivates silenced genes by epigenetically remodeling histones through inhibition of methylation and enhancement of acetylation, highlighting its therapeutic potential in BC (49). RSV significantly upregulates the expression of the ATP2A3 gene—by as much as sixfold—in both MCF-7 and MDA-MB-231 BC cell lines, contributing to apoptosis and alterations in intracellular calcium handling (50). Investigating the underlying epigenetic mechanisms, researchers found that RSV reduces histone deacetylase (HDAC) activity by approximately 50%, specifically lowering nuclear HDAC2 levels and its binding to the ATP2A3 promoter. This shift is associated with increased global histone H3 acetylation and enhanced enrichment of the activating H3K27Ac mark at the ATP2A3 promoter. While RSV also stimulated histone acetyltransferase (HAT) activity, inhibiting p300—a major HAT—did not significantly affect ATP2A3 expression, suggesting alternative acetyltransferases may be involved. Moreover, RSV reduced DNA methyltransferase (DNMT) activity and decreased expression of methyl-DNA binding proteins MeCP2 and MBD2, though the ATP2A3 promoter remained unmethylated in both cell lines. These findings highlight RSV's epigenetic modulation of ATP2A3 expression and support its potential as a therapeutic agent in BC (50).

### 4.3 Quercetin

A study investigated how quercetin influences cellular immunity against cancer by examining IL15 expression and its regulatory mechanisms (51). Results showed that quercetin treatment significantly reduced IL15 expression in both cell lines, with HeLa cells exhibiting approximately double the promoter methylation compared to controls, while A549 cells showed approximately three times the methylation level. The findings suggest that quercetin inhibits cancer cell proliferation by downregulating IL15 expression through increased promoter methylation (51). Another study explores the potential of quercetin, a dietary phytochemical, as a modulator of epigenetic pathways for anticancer strategies. The study quantified the activities of DNMTs, HDACs, HMTs, and global DNA methylation in HeLa cells treated with quercetin. Molecular docking studies indicated that quercetin interacts with the catalytic sites of DNMTs and HDACs, potentially functioning as a competitive inhibitor. Additionally, quantitative methylation arrays showed that quercetin downregulated global DNA methylation levels in a dose- and time-dependent manner. This led to a significant reduction in promoter methylation of tumor suppressor genes (TSGs) and restoration of their expression. The findings suggest that quercetin's ability to modulate chromatin modifiers and epigenetic markers could make it a promising candidate for epigenetic-based anticancer therapies (51) (Figure 2).

### 4.4 Vitamins

Although many researchers and healthcare professionals caution against high-dose vitamin supplements, particularly antioxidants, during cancer treatment, BC patients often continue to use these supplements. Given this, a cohort study worked on 4,877 Chinese women diagnosed with invasive BC and followed them for 4.1 years (52). They observed that vitamin use shortly before BC patients is linked to lower mortality and recurrence rates, even after adjusting for various lifestyle factors. Specifically, women who utilized antioxidants, such as vitamin E, vitamin C, and multivitamins experienced an 18% reduction in mortality risk and a 22% reduction in recurrence risk. These findings indicate that early supplementation may have a beneficial impact on long-term outcomes, although the effects can vary based on other treatment modalities, such as chemotherapy and radiotherapy. It is important for patients to consult with their healthcare providers before starting any new supplements to ensure they are appropriate for their individual health circumstances (52).

#### 4.4.1 Vitamin B

From a vitamin B point of view, Folate and other B vitamins related to methylation are crucial nutrients that significantly contribute to DNA synthesis, repair, and methylation processes. These functions suggest a possible link between these vitamins and the development of various types of cancer. A study tried to determine the serum vitamin B levels of 520 BC patients (53). They observed that serum levels of vitamin B1 and vitamin B5 in these patients and those with benign breast conditions were found to be higher than those of healthy individuals. In contrast, the expression levels of vitamin B3 were lower in BC patients compared to both the healthy control group and the benign breast disease group. Additionally, vitamin B1 levels showed a positive correlation with the risk of BC, while vitamin B3 levels exhibited a negative correlation with that risk. There was no significant relationship between vitamin B5 levels and BC risk (53). Another similar study also worked on 848 incident cases of invasive BC and found that plasma levels of folate, pyridoxal5′-phosphate (PLP), and vitamin B-12 were not linked to the overall risk of BC (54). However, higher plasma folate levels were moderately associated with an increased risk of premenopausal BC and with the development of estrogen receptor (ER)–positive or progesterone receptor (PR)–positive breast tumors. In contrast, a negative association was observed between plasma PLP levels and postmenopausal BC risk (54).

On the other hand, there are also some studies that tried to decrease the risk of BC with vitamin B supplement administration in BC patients. In this regard, Egnell et al. (55) investigated 462 BC patients and found that there is a relation between the intake of some B vitamins. For instance, dietary, supplemental, and total pyridoxine intakes, as well as total thiamin intake, were inversely associated with BC risk. However, they did not find any relation between the risk of BC and other members of the B vitamin family (55). Another study also assessed the relation between BC risk and vitamin B intake in Brazil (56). In this case–control study, 458 female patients aged 20–74 years with newly diagnosed and histologically confirmed invasive BC were included and genotyping of the three single-nucleotide polymorphisms (SNPs) in the *MTHFR* and *MTR* genes was performed on their blood samples (56). When women with the *MTR* 2756GG genotype had a significantly increased BC risk, they declared that a significant positive association can be observed between folate intake and BC risk in women with the MTHFR 1298AA, MTHFR 677CT and TT, and MTR 2756AA genotypes. Women with the lowest folate intake and the MTHFR 1298AC and CC genotypes exhibited an increased risk of BC. Conversely, those with the lowest folate intake and the MTHFR 677CT and TT genotypes showed a decreased risk. Statistically significant interactions were identified between folate intake and the MTHFR A1298C and C677T polymorphisms (57). They concluded that there are no overall significant associations between dietary intake of folate, vitamin B6, and vitamin B12, MTHFR genotype, and BC risk. However, an increased risk of BC was observed in women with the MTR 2756GG genotype, as well as a heightened risk linked to higher folate intake among premenopausal women, along with gene–nutrient interactions. Further research into these factors will be essential for understanding the etiology of BC (57). Another similar study in Japan also assessed the same polymorphisms in 388 BC patients (56). They also identified that the median dietary folate intake in the control group was 438.2 μg/day, with an interquartile range of 354.9 to 542.9. There were no significant associations found between the dietary intake of folate, vitamin B2, vitamin B6, or vitamin B12, nor between the polymorphisms of the MTHFR or MTR genes and BC risk. Additionally, no significant interactions were identified among the nutrients, genetic polymorphisms, and BCr risk. The associations between nutrients and BC risk did not vary based on hormone receptor status (56).

#### 4.4.2 Vitamin E

Vitamin E refers to a group of compounds that share similar yet distinct chemical structures and biological functions (58). Notably, certain vitamin E compounds have the intriguing ability to trigger apoptosis in cancer cells while sparing normal cells (58). In contrast, the parent compound RRR-α-tocopherol, commonly known as natural or authentic vitamin E and recognized for its antioxidant properties, does not promote apoptosis in cancer cells. Research aimed at understanding how specific forms of vitamin E can trigger apoptosis in cancer cells has revealed several non-antioxidant biological functions. These include the restoration of pro-apoptotic signaling pathways, such as transforming growth factor-β and Fas signaling (58). One of the first studies in this field was conducted in 1993 (59), which found that vitamin E demonstrates a dose-dependent protective effect on mononuclear leukocyte (MNL) DNA against oxidative damage caused by phagocytes. This seemingly novel protective function of vitamin E is primarily attributed to its ability to inhibit the production of ROS by activated neutrophils, rather than being linked to the traditional antioxidant scavenging properties of vitamin E (59). After this study, a great number of studies have confirmed the role of vitamin E in decreasing BC risk in women (60–63). One of the most recent studies in this field was conducted in 2023, which is a meta-analysis, and their findings revealed that vitamin E intake was inversely related to BC recurrence; however, no association was observed between vitamin E consumption and BC mortality (64).

### 4.5 Probiotics

Studies have shown that probiotics can help control cancer cell growth, particularly in gastrointestinal cancers. For example, *Lactobacillus rhamnosus* GG has been shown to suppress colon cancer cell proliferation and promote cell death. Research suggests that yogurt containing high levels of Lactobacillus may lower the risk of colorectal cancer. Probiotics may also help prevent cancer recurrence, as seen with *Lactobacillus casei* in bladder cancer (65). Prebiotics also offer cancer-fighting potential. When combined with probiotics (synbiotics), they may improve gut health and reduce cancer therapy side effects. Prebiotic supplements have been shown to boost immune function and beneficial gut bacteria in colorectal cancer patients. Overall, probiotics and prebiotics show promise in cancer prevention and treatment, especially in managing gut health. However, further research is needed to fully understand their role in cancer management (65).

### 4.6 Omega-3 fatty acids

In BC, besides many essential roles, PUFAs are able to affect epigenetic processes. To explore the mechanisms behind the cancer-protective effects of omega-3, Abbas and colleagues conducted a study using BALB/c mice and revealed genome-wide epigenetic changes in the F1 offspring of mothers who consumed diets rich in omega-3 fatty acids. Notably, they observed a significant increase in the acetylation of the H3K18 histone mark and a decrease in the H3K4me2 mark on nucleosomes near transcription start sites. These epigenetic alterations are linked to differential gene expression related to various pathways and molecular mechanisms that help prevent cancer, including the p53 pathway, G2M checkpoint, DNA repair, inflammatory response, and apoptosis (66). When the offspring mice were exposed to 7,12-Dimethylbenz(a)anthracene (DMBA), those whose mothers had a diet rich in omega-3 fatty acids exhibited delayed mortality, improved survival rates, reduced lateral tumor growth, and smaller tumor sizes. Notably, several genes, including BRCA genes, appeared to be epigenetically reprogrammed, positioning them for rapid transcriptional activation as a result of the maternal diet rich in canola oil (66). Another study tried to assess the impact of approximately 1 to 5 grams per day of eicosapentaenoic acid (EPA) and docosahexaenoic acid (DHA) over a 12-month period on the fatty acid and oxylipin profiles in breast adipose tissue among survivors of estrogen receptor and progesterone receptor-negative (ERPR(-)) BC (67). This study aims to compare the effects of approximately 5 grams per day vs. 1 gram per day of DHA and EPA supplementation in women who are within 5 years of finishing standard treatment for estrogen receptor and progesterone receptor-negative [ERPR (-)] BC, specifically in stages 0 to III. They observed that both dosage levels resulted in increased n-3 PUFAs in breast adipose tissue, erythrocytes, and plasma compared to baseline (67); however, the 5 grams per day supplement demonstrated greater efficacy, with differences of 0.76 in breast adipose, 6.25 in erythrocytes, and 5.89 in plasma. Additionally, the 5 g/d dosage led to a reduction in plasma triglycerides from baseline, with changes of 27.38 at 6 months and 24.58 at 12 months. The oxylipin profiles in breast adipose tissue exhibited dose-dependent increases in metabolites of DHA and EPA. Furthermore, distinct DNA methylation patterns observed in adipose tissue after 12 months suggest a potential downregulation of abnormal lipid metabolism pathways associated with the 5g/d dosage (67).

Another study tried to reveal the effects of PUFAs on lncRNAs. They worked on transgenic Fat-1 and wild-type C57BL/6J littermates and fed them with PUFAs (68). They observed that the Fat-1 group exhibits a significantly lower incidence of tumors and smaller tumor volumes compared to the control group. The n-3 polyunsaturated fatty acids (PUFAs) present in Fat-1 mothers alter the expression of long non-coding RNAs (lncRNAs) in the mammary glands of their offspring, with 53 lncRNAs being upregulated and 45 downregulated. These lncRNA alterations are linked to changes in mRNA levels across various oncogenic signaling pathways, particularly the NF-κB, Jak-STAT, and MAPK pathways. Key proteins associated with these pathways, including p65, p60, STAT3, Jak1, and p38, show significant inhibition in the Fat-1 group. Correspondingly, there is a reduction in cell proliferation and an increase in apoptosis observed in the mammary epithelial cells of the Fat-1 group compared to the control group (68).

## 5 Functional foods along with exercise help overcoming BC

**Figure 3:**
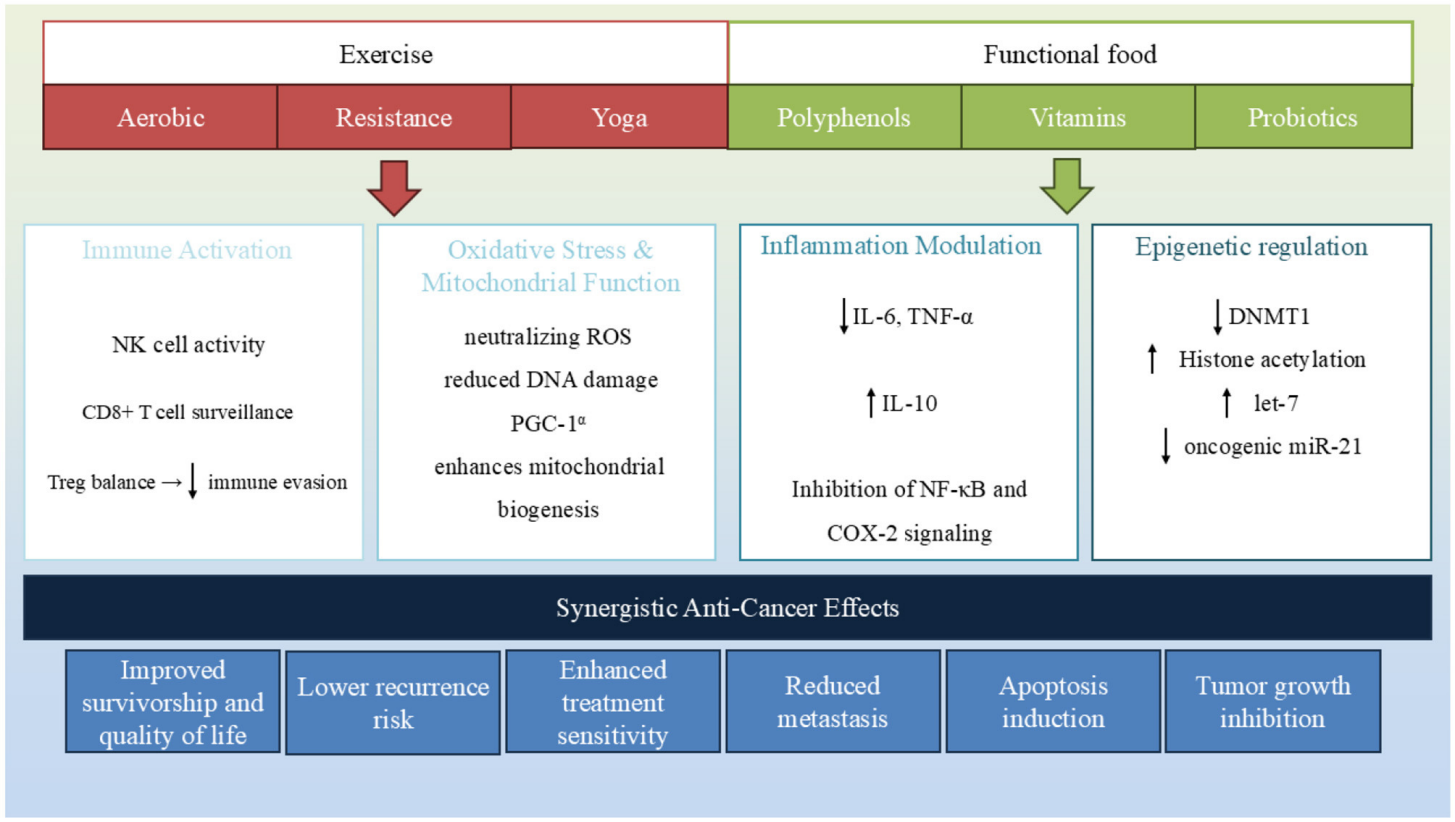
Functional foods and exercise converge on key biological pathways involved in breast cancer progression. Both interventions modulate epigenetic mechanisms, reduce inflammation and oxidative stress, enhance immune response, and regulate hormone levels. Their combined effect contributes to tumor suppression, improved therapy outcomes, and enhanced survivorship.

Functional foods and exercise are both powerful, non-pharmacological interventions that influence BC development and progression through overlapping molecular pathways. Increasingly, evidence suggests that these two lifestyle strategies may act synergistically, offering enhanced benefits when combined, particularly in modulating systemic inflammation, mitochondrial function, and epigenetic remodeling (Table 1, Figure 3).

**Table 1:** Summary of studies investigating the combined effect of functional foods and exercise in breast cancer.

**Functional food component**	**Study model**	**Intervention type**	**Key outcomes**	**Reference**
Vitamin D	Human	vitamin D (4,000 IU) +yoga	Body fat percentage, handgrip strength, and QoL indicators, including global health, functional scales, and symptom scales, showed significant improvements and reductions in TNF-α and interleukin-6 levels	(69)
	*In vivo*	interval exercise training (IET) and vitamin D	Vitamin D alone increased tumor growth by approximately 23%, whereas its combination with exercise reduced tumor growth by 12%	(70)
Probiotics	Human	body balance isometric exercises+ high protein diet containing pro- and prebiotics	significant reductions in weight and fat. Diet+Exercise group achieved losses of 6.5 kg, 3.3% BF, and 2% visceral fat	(71)
	Human	prebiotic fiber supplementation and Alberta Cancer Exercise	cancer survivors showed minimal changes in gut microbiota following aerobic exercise (ACE)	(73)
	*In vivo*	prebiotic fiber supplementation and Alberta Cancer Exercise	mice exhibited a consistent decrease in tumor volume over time when colonized with post-exercise microbiota. favorable cytokine profiles, including lower levels of VEGF	(73)
Omega-3 fatty acid	Human	12-week nutrition and exercise education+3g/d of long chain omega 3 fatty acid	changes in LBM using air displacement plethysmography, and quality of life, inflammation markers, fatigue, physical activity, menopausal symptoms, dietary intake, joint pain, and function indices	(75)
	Human	omega-6 (O6-PUFA) vs. omega-3 (O3-PUFA) fatty acid supplementation	O6-PUFA supplementation led to a statistically significant reduction in CRF compared to O3-PUFA	(76)

### 5.1 Vitamins

As mentioned before, vitamin supplements are widely used in BC patients as antioxidants.

*Vitamin D*. Naderi and colleagues in 2022 (69) used the combination of yoga training with high vitamin D dose supplementation on BC survivors and examined the expression and systemic levels of inflammatory cytokines in their serum. Volunteers were randomly assigned to one of three groups: a high dose (4,000 IU) vitamin D supplementation group (HD) with 10 participants, a yoga group receiving a high dose of vitamin D (YHD) with 10 participants, and a yoga group with a low dose (2,000 IU) of vitamin D (YLD) also consisting of 10 participants. All participants engaged in Hatha yoga sessions twice a week for 12 weeks (69). Their results show that some measures including body fat percentage, handgrip strength, and QoL indicators, such as global health, functional scales, and symptom scales, showed significant improvements in both the YHD and YLD groups when compared to the HD group. Additionally, interleukin-10 (IL-10) levels were significantly higher in the Y-HVD group compared to the Y-LVD and HVD groups. Furthermore, there were notable reductions in tumor necrosis factor-α (TNF-α) and interleukin-6 levels in the Y-HVD group following the intervention. The anti-inflammatory index (IL-10/TNF-α) also significantly increased in both yoga groups. This study shows that the yoga training increases the effects of vitamin D supplements (especially the high dosages of it) on the inflammatory response of BC. However, due to the small sample size of this study, further investigations are needed for confirmation (69). Another study investigates the combined impact of interval exercise training (IET) and vitamin D supplementation on BC progression in mice (70). Researchers divided female Naval Medical Research Institute (NMRI) mice into five groups: healthy control, cancer control, cancer with vitamin D, cancer with exercise, and cancer with both interventions. Findings revealed that vitamin D alone increased tumor growth by approximately 23%, whereas its combination with exercise reduced tumor growth by 12% compared to the cancer control group. Additionally, the group receiving both treatments exhibited the highest total antioxidant capacity and significant upregulation of genes associated with mitochondrial function in cardiac tissue. These results suggest that while vitamin D alone may promote tumor growth, its combination with exercise can counteract this effect, enhancing antioxidant defenses and mitochondrial health. The study underscores the potential of integrating exercise with vitamin D supplementation as a strategy to manage BC progression and improve cardiac function (70).

### 5.2 Probiotic

Artene and colleagues (71) worked on 165 ER+/PR±/HER2-luminal A and B BC patients after surgery and chemotherapy, on antiestrogenic medication. They used a kind of high-protein diet containing pro- and prebiotics. They also used body balance isometric exercises on one of the groups of patients. They observed that the 1-year outcomes for the patients indicate that both the Diet+Exercise and Diet groups experienced statistically significant reductions in weight and fat. However, the Diet group did not show statistically significant fat loss over the entire intervention period. When comparing the initial measurements to those taken at the 12-month mark, the Diet group lost 3.3 kg, 3.2% body fat (BF), and 1% visceral fat, while the Diet+Exercise group achieved losses of 6.5 kg, 3.3% BF, and 2% visceral fat (71). After BC diagnosis, weight gain is common and significantly impacts patient outcomes, as it is associated with higher risks of recurrence, second primary tumors, cardiovascular disease, and decreased overall survival. Biological mechanisms driving this include increased inflammation, hyperinsulinemia, changes in adipokines, and elevated estrogen levels from excess fat tissue (72).

Weight management plays a critical role in the prognosis of BC patients. According to the article, post-diagnosis weight gain is linked to increased risks of recurrence, second primary cancers, and overall mortality (72). Excess adiposity promotes inflammation, insulin resistance, and hormonal imbalances that can fuel tumor growth. Evidence suggests that even modest intentional weight loss improves metabolic health markers and may lower cancer-related risks. Therefore, integrating supervised weight loss programs that focus on diet quality, physical activity, and behavioral support is increasingly recommended as a core component of survivorship care. Given this, the study by Artene et al. (71) is important in the field of BC; however, a great weakness of this study is that it does not reveal the type of probiotics used on patients. From a prebiotic point of view, Sampsell and colleagues (73) worked on both BC patients and mice and used the combination of prebiotic fiber supplementation and Alberta Cancer Exercise (ACE) program. According to their study, while cancer survivors showed minimal changes in gut microbiota following aerobic exercise (ACE), mice exhibited a consistent decrease in tumor volume over time when colonized with post-exercise microbiota than pre-exercise microbiota, with significant differences observed on days 16 and 22. Beta diversity analysis indicated that the injection of EO771 breast tumor cells and treatment with Paclitaxel chemotherapy modified the gut microbial communities in the mice. The post-exercise microbiota groups displayed an enrichment of potentially protective microbes. Mice with post-exercise microbiota also showed more favorable cytokine profiles, including lower levels of vascular endothelial growth factor (VEGF). The positive microbial and molecular effects were further enhanced by prebiotic supplementation. Both exercise and prebiotic fiber appeared to have an adjuvant effect, likely through an improved anti-tumor immune response influenced by beneficial shifts in gut microbiota (73).

### 5.3 Omega-3 fatty acids

As mentioned before, omega-3 is widely used for different aspects of BC patients. The combination of exercise and omega-3 is mostly used for one the following side effects in BC patients: fatigue, muscle weakness, and loss of lean body mass (LBM). After undergoing treatment for BC, many women experience notable increases in body weight (74). Unfortunately, these changes often involve a simultaneous decrease in LBM and an increase in fat tissue. Notably, such shifts in body composition can also occur without any noticeable change in overall body weight. Research from BC populations has identified several factors strongly linked to weight gain, including being premenopausal at diagnosis, undergoing treatment-induced menopause, having a lower body weight at diagnosis, reduced physical activity levels, and receiving prolonged chemotherapy. Pharmacological studies suggest that starting or switching to aromatase inhibitors from tamoxifen may lead to increases in LBM, likely due to changes in sex hormone levels (74). Although the exact reasons for LBM loss in this group remain unclear, it has been associated with poorer metabolic health, including a heightened risk of cardiovascular disease and metabolic syndrome-related conditions. At present, there are no conclusive guidelines regarding the ideal body weight or weight change targets for women who have completed BC treatment. However, epidemiological evidence suggests that maintaining a stable weight might be linked to improved survival outcomes.

In this regard, a study group tried to prevent LBM through the use of omega-3 and exercise (75). A total of 153 women who have finished BC treatment had a body mass index (BMI) ranging from 20 to 35 kg/m^2^ were divided into three groups: one receiving 3 g/d of long chain omega-3 fatty acid [LCn-3s (N-3)], another participating in a 12-week nutrition and exercise education program along with olive oil (P-LC), and the third group engaging in the education program combined with LCn-3s (EX+N-3). Their education program included nine sessions over 12 weeks, focusing on healthy eating and supervised resistance exercise circuits, with additional advice on regular resistance and aerobic training. Primary outcomes of this study were changes in LBM using air displacement plethysmography, while secondary outcomes included quality of life, inflammation markers, fatigue, physical activity, menopausal symptoms, dietary intake, joint pain, and function indices (75).

In a multicenter randomized controlled trial, researchers investigated the effects of omega-6 (O6-PUFA) vs. omega-3 (O3-PUFA) fatty acid supplementation on cancer-related fatigue (CRF) among BC survivors (76). Contrary to expectations, the study found that O6-PUFA supplementation led to a statistically significant reduction in CRF compared to O3-PUFA. Specifically, participants receiving O6-PUFA experienced a mean decrease of 2.51 points in fatigue scores over 6 weeks, whereas those on O3-PUFA saw a 0.93-point reduction. The most pronounced benefits were observed in individuals with severe baseline fatigue (scores ≥7) (76). Additionally, O6-PUFA supplementation was associated with significant decreases in proinflammatory markers, particularly within the TNF-α signaling pathway, suggesting a potential anti-inflammatory mechanism underlying the fatigue reduction. These findings challenge the traditional view of omega-6 fatty acids as proinflammatory and highlight the need for further research to elucidate their role in managing CRF among BC survivors (76, 77). In another aspect, a meta-analysis of over 274,000 women revealed that a higher dietary omega-3 to omega-6 (n−3/n−6) ratio is associated with a 10% reduction in BC risk. Increasing the intake of omega-3 fatty acids, particularly from marine sources, while decreasing omega-6 fatty acids, can be beneficial. Additionally, consuming polyphenol-rich foods, such as those found in the Mediterranean diet, can enhance the n-3/n-6 ratio and further reduce BC risk. Organic foods tend to have higher levels of polyphenols and a more favorable n-3/n-6 ratio compared to conventional foods. However, we could not find any evidence in field; therefore, we suggest that future studies should focus on the effects of exercise on n-3/n-6 ratio (78).

In summary, both functional foods and exercise converge on shared biological processes:

I. Inflammation Modulation: Polyphenols, omega-3 fatty acids, and vitamin D suppress pro-inflammatory cytokines, such as IL-6 and TNF-α. Exercise similarly activates anti-inflammatory signaling via myokines, creating a complementary effect that may reduce tumor-promoting inflammation.

II. Oxidative Stress and Mitochondrial Function: Antioxidant-rich functional foods (e.g., vitamins C and E and sulforaphane) reduce reactive oxygen species (ROS), while aerobic exercise enhances mitochondrial biogenesis and oxidative metabolism. Together, these interventions may reduce oxidative DNA damage and support apoptosis.

III. Epigenetic Regulation: Several dietary compounds influence epigenetic markers—folate and EGCG impact DNA methylation, curcumin and resveratrol affect histone acetylation, and vitamin D modulates miRNA expression. Exercise, too, alters DNA methylation in tumor-suppressor and inflammatory genes, suggesting the potential for reinforced epigenetic reprogramming when both are combined.

IV. Immune and Hormonal Modulation: Functional foods and exercise enhance immune surveillance and reduce circulating estrogens, critical in hormone-sensitive BC subtypes.

To validate these findings, future work should focus on randomized controlled trials testing integrated lifestyle interventions using functional foods and exercise, with endpoints such as recurrence, inflammation, and quality of life and personalized, omics-guided strategies to match specific dietary and physical activity regimens to individual epigenetic or metabolic profiles. Together, these approaches will help clarify how lifestyle-based synergy can be harnessed in BC prevention and care.

## 6 Modulation of programmed cell death by functional foods along with exercise

### 6.1 RSV

In studies using 4T1 murine BC cells, RSV demonstrated clear dose-dependent cytotoxicity (79). In human BC MCF-7 cells, which lack functional caspase-3, RSV still induced substantial apoptosis, though through a caspase-independent mechanism (80). Despite the absence of typical apoptosis indicators such as cytochrome c release, caspase activation, and PARP cleavage, mitochondrial membrane potential was significantly disrupted. A rapid drop in mitochondrial potential was observed after RSV exposure, implicating mitochondrial dysfunction as an upstream event in apoptosis. Importantly, this effect was mitigated in cells overexpressing Bcl-2, underlining the role of the mitochondrial pathway. Alongside mitochondrial disruption, there was a marked increase in ROS and nitric oxide (NO) levels, suggesting oxidative stress as a critical mediator of cell death (80). At the signaling level, RSV was found to inhibit Akt phosphorylation, thereby reducing a major pro-survival signal within cancer cells. It also disrupted the redox balance by depleting glutathione levels and increasing lipid peroxidation. These oxidative events activated stress-related MAP kinases, including JNK (early phase) and p38 MAPK (later phase), both of which are known to promote apoptotic signaling under oxidative conditions (81). *In vivo*, RSV's efficacy was confirmed in mouse models bearing MDA-MB-231 xenografts. Treated tumors showed reduced volume, diminished vascularization, and a higher proportion of apoptotic cells. These effects were particularly pronounced in ERα-negative/ERβ-positive tumors. Consistently, RSV also reduced VEGF secretion *in vitro*, highlighting its dual role in triggering apoptosis and suppressing angiogenesis (82).

### 6.2 Quercetin

One notable study evaluated a vanadium–quercetin complex for its chemotherapeutic potential against chemically induced BC in rats and MCF-7 human BC cells (83). Characterization using spectroscopic techniques confirmed the successful synthesis of the complex. This compound exhibited strong antioxidant properties and induced apoptosis in a dose-dependent manner. Molecular analyses showed upregulation of p53 and caspases 3 and 9, along with downregulation of Akt, mTOR, and VEGF expression, implying the involvement of intrinsic apoptotic pathways. *In vivo*, histological assessment revealed significant reversal of hyperplastic lesions. The TUNEL assay demonstrated an increase in apoptosis, and immunohistochemistry confirmed decreased cell proliferation in treated groups. Together, these findings highlight the therapeutic potential of the vanadium–quercetin complex in BC treatment (83). Furthermore, *in vitro* data corroborated quercetin's ability to inhibit the proliferation of MCF-7 cells in a dose- and time-dependent manner. While low concentrations had minimal effect, higher concentrations significantly suppressed proliferation, with the highest dose yielding up to 85% inhibition. This antiproliferative effect was closely linked to the modulation of Bcl-2 and Bax proteins: Bcl-2 expression was significantly reduced while Bax levels increased, reinforcing quercetin's pro-apoptotic effect through mitochondrial disruption (84). Treatment with doses ranging from 10 to 175 μM led to over 90% reduction in MCF-7 cell viability. At higher doses, S-phase arrest was evident, accompanied by suppressed expression of CDK2, cyclins A and B, and increased levels of tumor suppressor proteins, such as p53 and p57. Apoptosis was mediated via caspase activation and mitochondrial pathways, indicated by loss of membrane potential and increased AIF translocation from mitochondria to the nucleus. Additionally, quercetin triggered the release of GADD153 from the endoplasmic reticulum to the nucleus, suggesting involvement of ER stress in cell death (85). In MDA-MB-231 cells, quercetin was shown to regulate apoptosis and cell cycle progression through the JNK-Foxo3a signaling axis. Knockdown of Foxo3a via siRNA markedly diminished quercetin-induced apoptosis and cell cycle arrest, as evidenced by reduced S and G2/M phase populations. Similarly, inhibition of JNK—known to regulate Foxo3a—nullified the increase in these transcriptional activities, confirming JNK-Foxo3a as a critical upstream mediator in quercetin's pro-apoptotic mechanism (86). Importantly, the combination of quercetin administration with exercise training has demonstrated synergistic benefits in models of metabolic disease with implications for cancer prevention and progression. In a study involving diabetic obese rats, HIIT and moderate-intensity continuous training (MICT) were combined with quercetin supplementation (15 mg/kg) over an 8-week period. Both training protocols alone and in combination with quercetin improved blood glucose levels, lipid profiles, and inflammatory markers. Molecular analysis revealed that these interventions preserved PI3K, AKT, and FOXO3 protein levels and enhanced caspase-8 gene expression in cardiac tissue, indicating protective effects against diabetic cardiomyopathy. These findings support the hypothesis that quercetin, particularly when paired with exercise, may exert systemic anti-inflammatory and pro-apoptotic effects through modulation of the PI3K/AKT/FOXO3 pathway (87).

### 6.3 Saffron

In a study investigating the impact of crocin on BT-474, a HER2+ BC cell line, crocin significantly reduced cell viability and triggered apoptosis through caspase-9 activation and cleavage. Additionally, crocin induced splicing of the XBP1 gene, suggesting involvement of the unfolded protein response (UPR) in its pro-apoptotic mechanism (88). Beyond *in vitro* studies, saffron's effectiveness in combination with exercise training has been explored in BC-bearing mice. One investigation assessed the effects of HIIT and saffron aqueous extract (SAE), both individually and combined, on tumor progression and apoptotic markers in mice injected with 4T1 BC cells. Both HIIT and SAE alone significantly reduced tumor volume and increased caspase-3 and Bax protein levels, while decreasing Bcl-2 levels, indicating enhanced apoptotic activity. However, the combination of HIIT and SAE did not show a synergistic benefit. Instead, the HIIT+SAE group had lower Bax and caspase-3 levels and a reduced Bax/Bcl-2 ratio compared to either treatment alone, suggesting that the combination may attenuate the pro-apoptotic effects seen with individual treatments (89). These effects were evidenced by decreased Bax and caspase-3 levels and elevated Bcl-2 expression in both HIIT and SAE groups. However, these benefits were not observed when the two interventions were combined. The HIIT+SAE group did not significantly improve apoptotic indices or counteract weight loss compared to controls, reinforcing the conclusion that combined administration may diminish the individual efficacy of saffron and exercise in this model (90).

### 6.4 Seeds

Seeds from various plants have been traditionally used for their medicinal properties, and recent studies have begun to provide scientific evidence for their apoptotic and anticancer effects in BC. One such example is *Lepidium sativum* (garden cress), whose aqueous seed extract was evaluated for its cytotoxic effects on MCF-7 BC cells and normal human fibroblasts. Despite some apoptosis also being noted in fibroblasts, morphological and staining analyses indicated that *Lepidium sativum* exerted greater apoptotic effects on MCF-7 cells. Notably, the extract appeared to induce apoptosis independently of caspase-3, a protein lacking in MCF-7 cells, suggesting activation of alternative apoptotic pathways (91). Another promising candidate is *Nigella sativa* (black seed), a plant widely recognized for its therapeutic applications. In a novel investigation, seed proteins from *Nigella sativa* were isolated, partially purified, and tested for cytotoxicity in MCF-7 BC cells. Two protein fractions (P1 and P4) exhibited potent antiproliferative activity with IC50 values of 14.25 μg/mL and 8.05 μg/mL, respectively. Gene expression analysis indicated that the mechanism of cell death involved apoptosis, and protein identification through LC-MS/MS revealed various cytosolic proteins involved in metabolic processes. These findings suggest that proteins, not just the commonly studied thymoquinone, may contribute significantly to the anticancer potential of Nigella sativa (92). Grape seed proanthocyanidins (GSPs) have also demonstrated significant anticancer activity against multiple BC cell lines. *In vitro*, GSPs inhibited proliferation and induced apoptosis in 4T1, MCF-7, and MDA-MB-468 cells in a dose- and time-dependent manner. Mechanistically, GSPs enhanced the Bax/Bcl-2 ratio, promoted cytochrome c release, activated Apaf-1, and stimulated caspase-3 and PARP cleavage. Importantly, knockdown of Bax expression blocked GSP-induced apoptosis, confirming the pivotal role of the intrinsic mitochondrial pathway. *In vivo*, dietary GSPs reduced tumor growth and lung metastasis in 4T1 tumor-bearing mice and improved overall survival. These results underscore the ability of GSPs to trigger both caspase-dependent and -independent apoptotic pathways and inhibit metastatic progression (93).

Phoenix dactylifera (date palm) seeds have also been explored for their anticancer potential. Ethanolic extract of Phoenix dactylifera seed (PDSE) inhibited the proliferation of MDA-MB-231 and MCF-7 BC cells, with maximum effect seen in triple-negative MDA-MB-231 cells (IC50 = 85.86 μg/mL), while sparing normal cells. PDSE induced loss of mitochondrial membrane potential, promoted late apoptosis, and arrested the cell cycle in the S phase. Western blot analysis showed activation of caspase-3 and PARP cleavage, alongside upregulation of Bax and downregulation of Bcl-2. *In silico* docking confirmed strong binding affinity of PDSE-derived rutin and quercetin with caspase-3, further supporting their role in apoptosis induction (94). Finally, *Mangifera pajang* (a lesser-known mango species) kernel extract showed cytotoxicity in both hormone-dependent (MCF-7) and triple-negative (MDA-MB-231) BC cells, with IC50 values of 23 μg/mL and 30.5 μg/mL, respectively. The extract triggered sub-G1 cell cycle arrest indicative of apoptosis, and flow cytometry confirmed early apoptotic events. Caspase-2,−3, and−9 appeared to mediate this cell death, with different caspase dependencies in each cell line. These findings suggest that M. pajang kernel extract activates both intrinsic and extrinsic apoptotic cascades, positioning it as a potential source of anti-cancer agents (95). In summary, seed-derived phytochemicals and proteins from *Lepidium sativum, Nigella sativa, Phoenix dactylifera, Mangifera pajang*, and grape seeds show considerable promise in inducing apoptosis in BC cells. These seeds act through diverse mechanisms involving mitochondrial pathways, caspase activation, and DNA fragmentation, with minimal toxicity to normal cells. Their inclusion in dietary strategies or development into novel therapeutics could offer safer and more targeted treatments for various BC subtypes.

### 6.5 Luteolin and genistein flavonoids

Luteolin, a flavonoid abundant in various fruits, vegetables, and medicinal herbs, has garnered attention for its potent anticancer properties, particularly in BC. Several studies have elucidated its mechanisms of inducing apoptosis and inhibiting tumor growth across different BC cell lines. In MDA-MB-231 cells, luteolin demonstrated a robust, dose-dependent inhibitory effect on proliferation and invasion. It induced cell cycle arrest at the S phase and significantly enhanced apoptosis. Mechanistically, luteolin suppressed telomerase activity by downregulating human telomerase reverse transcriptase (hTERT), a key component of the telomerase enzyme complex. This suppression was mediated via inhibition of the NF-κB signaling pathway and downregulation of c-Myc, a transcriptional activator of hTERT. These findings suggest that luteolin may exert its antitumor effect in part by targeting telomere maintenance mechanisms (96). Additionally, luteolin has shown potential as an adjuvant in chemotherapy. When co-administered with paclitaxel in MDA-MB-231 cells, luteolin not only enhanced paclitaxel-induced apoptosis but also activated the extrinsic apoptotic pathway. This was evidenced by the upregulation of Fas and activation of caspase-8 and caspase-3. The enhanced Fas expression was linked to inhibition of STAT3, a transcription factor often constitutively active in cancers. Importantly, this combination therapy led to significant tumor volume and weight reduction *in vivo*, indicating a promising strategy for combinatorial treatment (97). In hormone receptor-positive MCF-7 BC cells, luteolin inhibited proliferation through both extrinsic and intrinsic apoptotic pathways. It triggered cell cycle arrest at the sub-G1 and G1 phases and enhanced the expression of death receptor DR5. Activation of caspases −8, −9, and −3, along with PARP cleavage, was observed, suggesting a synergistic activation of both apoptotic routes. Mitochondrial dysfunction was also noted, with a decrease in mitochondrial membrane potential and release of cytochrome c, concurrent with increased Bax and decreased Bcl-2 expression (98). Collectively, these findings position luteolin as a multifunctional agent capable of targeting diverse molecular pathways to promote apoptosis in BC.

Genistein, a soy-derived isoflavone with estrogen-like properties, has also been extensively studied for its anticancer effects. Its consumption has been associated with a lower incidence of BC in Asian populations, possibly due to dietary habits rich in soy products. In MCF-7 cells, genistein suppressed proliferation and induced apoptosis by modulating the IGF-1R-PI3K/Akt pathway. Treatment led to downregulation of IGF-1R and phosphorylated Akt, which in turn decreased the Bcl-2/Bax ratio at both the protein and mRNA levels. This shift toward pro-apoptotic signaling suggests that genistein disrupts survival pathways critical for cancer cell maintenance (99). In MDA-MB-231 triple-negative BC (TNBC) cells, genistein also induced apoptosis independent of the p53 pathway. It upregulated pro-apoptotic Bax and cell cycle regulator p21WAF1 while downregulating Bcl-2 and p53. Apoptosis was confirmed through classical hallmarks, such as DNA fragmentation, activation of CPP32 (caspase-3), and cleavage of PARP. Flow cytometry analysis supported a time-dependent increase in apoptotic cells, highlighting genistein's pro-apoptotic activity in a p53-independent manner (100). Another study in MDA-MB-231 cells further identified the MEK5/ERK5/NF-κB axis as a critical regulatory mechanism targeted by genistein. It was shown to suppress MEK5 and ERK5 signaling, leading to decreased NF-κB DNA-binding activity and subsequent downregulation of Bcl-2. Concomitantly, Bax expression and caspase-3 activation were increased, suggesting that genistein mediates apoptosis by disrupting survival signaling and promoting caspase activation through mitochondrial pathways (101).

## 7 Functional food and chemotherapy tolerance and side effects

Functional foods, which are defined as foods that provide health benefits beyond basic nutrition, have garnered attention for their potential role in enhancing chemotherapy tolerance and mitigating side effects in cancer patients (also summarized in Table 2). Chemotherapy, while effective in targeting cancer cells, often comes with a range of adverse effects, including nausea, fatigue, and compromised immune function. Curcumin is one of the functional foods that can decrease the chemotherapy side effects and thereby increase the quality of life for BC patients (102). After 21 days of turmeric supplementation, there was a statistically significant improvement in overall health status. However, scores on the functional domains—physical, role, emotional, and cognitive—did not show notable changes before and after the intervention, although a decrease in social functioning was observed. Noteworthy improvements were seen in symptoms commonly associated with cancer, including fatigue, nausea and vomiting, pain, insomnia, appetite loss, constipation, and diarrhea. Only three participants reported experiencing mild dyspnea. Additionally, there was no significant change in participants' perceived financial burden based on questionnaire results after the turmeric intake period (102).

**Table 2:** Studies investigating the effects of different functional foods in reducing the side effects of chemotherapy.

**Side effect**	**Drug**	**Type of functional food**	**Study model**	**Dose**	**Administration**	**Results**	**References**
Fatigue	Paclitaxel	Curcumin	human	2 g/day for 21 days	Oral	There was a significant difference in the scores of cancer-related symptoms, such as fatigue	(102)
	-	Curcumin	Human	1 gm/day	Oral	Significant reduction in fatigue in the curcumin group	(104)
	-	Omega-3 and Vit D	Human	300 mg ω3 capsules daily, and one 50,000IU VitD tablet weekly,	Oral	Lower scores for fatigue	(105)
	-	probiotics + prebiotics	Human	twice a day for 8 weeks	oral	Fatigue decreased significantly in the synbiotics group compared to the placebo group	(108)
GI side effects	Paclitaxel	Curcumin	human	2 g/day for 21 days	Oral	There was a significant difference between pre- and post-intervention groups in nausea and vomiting, appetite loss, constipation, and diarrhea	(102)
	-	Curcumin	Human	1 gm/day	Oral	Significant reduction in nausea and diarrhea in the curcumin group	(104)
	-	Omega-3 and Vit D	Human	300 mg ω3 capsules daily, and one 50,000IU VitD tablet weekly,	Oral	lower scores for nausea and vomiting, and appetite loss	(105)
	-	probiotics + prebiotics	Human	twice a day for 8 weeks	oral	Abnormal defecation decreased significantly in the synbiotics group compared to the placebo group.	(108)
Pain	Paclitaxel	Curcumin	human	2 g/day for 21 days	Oral	There was a significant difference between pre- and post-intervention groups in pain	(102)
	aromatase inhibitors	hydroxytyrosol, omega-3 fatty acids, and curcumin	human	for 1 month	Oral	Pain scores decreased during the therapy due to decreased inflammation	(103)
	-	Curcumin	Human	1 gm/day	Oral	Significant reduction in pain in the curcumin group	(104)
	-	Omega-3 and Vit D	Human	300 mg ω3 capsules daily, and one 50,000IU VitD tablet weekly,	Oral	Lower scores for pain	(105)
Insomnia	Paclitaxel	Curcumin	human	2 g/day for 21 days	Oral	There was a significant difference between pre- and post-intervention groups in insomnia	(102)

A prospective, multicenter, open-label, single-arm clinical trial included 45 postmenopausal women with BC who exhibited elevated C-reactive protein (CRP) levels and were primarily receiving aromatase inhibitors. Participants were given a 1-month regimen consisting of hydroxytyrosol, omega-3 fatty acids, and curcumin. They found that pain levels showed a noticeable decline throughout the treatment, and no serious side effects were reported. They concluded that this supplement combination of hydroxytyrosol, omega-3 fatty acids, and curcumin effectively reduced inflammation, as evidenced by lower CRP levels, and relieved pain associated with aromatase inhibitor-related musculoskeletal symptoms. Furthermore, large-scale, long-duration clinical trials are needed to compare this regimen with conventional anti-inflammatory treatments and to evaluate its impact on clinical outcomes (103). Another study recruited patients diagnosed with locally advanced or metastatic BC. Participants received standard chemotherapy for 4 to 6 cycles, while curcumin was administered as an oral adjunct therapy at a dose of 1 gram per day, divided into two doses taken after breakfast and dinner. The primary goal of the study was to evaluate adverse events and treatment efficacy in these patients (104).

Another study aimed to evaluate the impact of omega-3 fatty acids and vitamin D supplementation on QoL and inflammatory biomarkers—tumor necrosis factor-alpha (TNF-α) and high-sensitivity C-reactive protein (hsCRP)—in women newly diagnosed with BC (105). Eighty-eight women with newly diagnosed BC were randomly assigned to one of four groups: (i) ω3 supplementation, (ii) VitD supplementation, (iii) combined ω3+VitD supplementation, or (iv) control. The interventions lasted 9 weeks, during which participants received either two 300 mg ω3 capsules daily, one 50,000 IU VitD tablet weekly, or both. After 9 weeks, participants in the combined ω3+VitD group showed significant improvement in overall global health than other groups. This group also reported better functional scores and reduced symptoms, including fatigue, nausea, vomiting, pain, and appetite loss. Notably, there were significant intergroup differences in TNF-α and hsCRP levels. A marked reduction in hsCRP was observed in the ω3 group compared to the control, while the ω3+VitD group experienced significant decreases in both hsCRP and TNF-α. No significant changes were observed in inflammatory markers for the ω3 or VitD groups alone (105).

Another study used women with metastatic BC who were receiving stable systemic therapy and were randomized in a 2:1 ratio to either an 8-week whole-food, plant-based (WFPB) dietary intervention group or a usual care control group. The intervention group attended weekly educational sessions and followed an ad libitum WFPB diet, with three prepared meals provided daily. A total of 20 participants in the intervention group and 10 in the control group completed the study. Fatigue severity and peak fatigue, measured by the Brief Fatigue Inventory (BFI), significantly decreased within the WFPB group. They concluded that adopting a whole-food, plant-based diet is achievable for women with MBC and may lead to improvements in QoL and treatment-related symptoms such as fatigue and cognitive function. Further research is recommended to validate these findings (106). From a vitamins point of view, a double-blind, randomized, placebo-controlled study assessed whether strength training (ST) combined with vitamins C and E could influence perceived and performance fatigability in BC survivors. Twenty-five participants were assigned to either a vitamin supplementation group or a placebo group. Both groups underwent a 10-week ST program, training twice weekly. The VIT group received daily doses of 500 mg vitamin C and 180 mg vitamin E, while the PLA group received 1 g/day of polydextrose. Fatigue was evaluated at baseline and after the intervention using the Multidimensional Fatigue Inventory (MFI)-20, and performance fatigability was assessed via 30 maximal isokinetic knee extensions. Results indicated similar reductions in general and physical fatigue across both groups, with no significant between-group differences (p > 0.05). Performance fatigability also improved comparably in both groups. The findings suggest that while ST is effective in reducing fatigue among BCS, the addition of antioxidant vitamins C and E offers no additional benefit (107). A clinical trial about synbiotics included 67 women with a confirmed diagnosis of BC who were admitted for single-day chemotherapy sessions. Participants were randomly assigned to receive either a synbiotic supplement (intervention group) or a placebo (control group) over an 8-week period. The synbiotic was administered orally twice daily. After 8 weeks and adjusting for potential confounding factors, patients in the synbiotic group experienced a significant reduction in chemotherapy-induced fatigue (p < 0.001) and abnormal bowel movements compared to the placebo group. Although there were improvements in nausea/vomiting and anorexia from baseline in the synbiotic group, these changes were not statistically significant when compared to the control group (108).

## 8 Limitations and future perspectives

Despite growing evidence supporting the epigenetic impact of exercise on BC prevention and management, several limitations remain in the current body of research. First, much of the existing data stems from animal models or *in vitro* studies, which may not fully capture the complexity of human physiology. Human studies often vary in design, with differences in exercise type, intensity, duration, and population characteristics, making it difficult to establish standardized protocols or draw definitive conclusions. Furthermore, many studies measure epigenetic changes in peripheral blood or muscle tissue, which may not accurately reflect modifications occurring in breast tissue or tumor cells. There is also a lack of longitudinal studies that track epigenetic changes over time in response to consistent exercise regimens, limiting our understanding of the long-term stability and reversibility of these modifications. In addition, while correlations between exercise and beneficial epigenetic outcomes have been observed, causality remains difficult to establish. It is also unclear whether specific subtypes of BC respond differently to exercise-induced epigenetic changes, which is critical for tailoring personalized interventions. Looking ahead, future research should focus on large-scale, well-controlled clinical trials to determine optimal exercise prescriptions (frequency, duration, and type) for epigenetic benefit. Advancements in high-throughput epigenomic technologies and single-cell sequencing will enhance our ability to detect subtle, cell-specific changes in response to exercise. Additionally, integrating multi-omics approaches—combining genomics, epigenomics, transcriptomics, and metabolomics—will provide a more comprehensive understanding of the biological pathways involved. Ultimately, interdisciplinary collaboration among oncologists, exercise physiologists, and molecular biologists will be essential in translating these findings into clinical practice. Personalized, evidence-based exercise interventions hold great promise as adjunct therapies in BC care, but more rigorous research is necessary to unlock their full potential.

## 9 Conclusion

**Figure 4:**
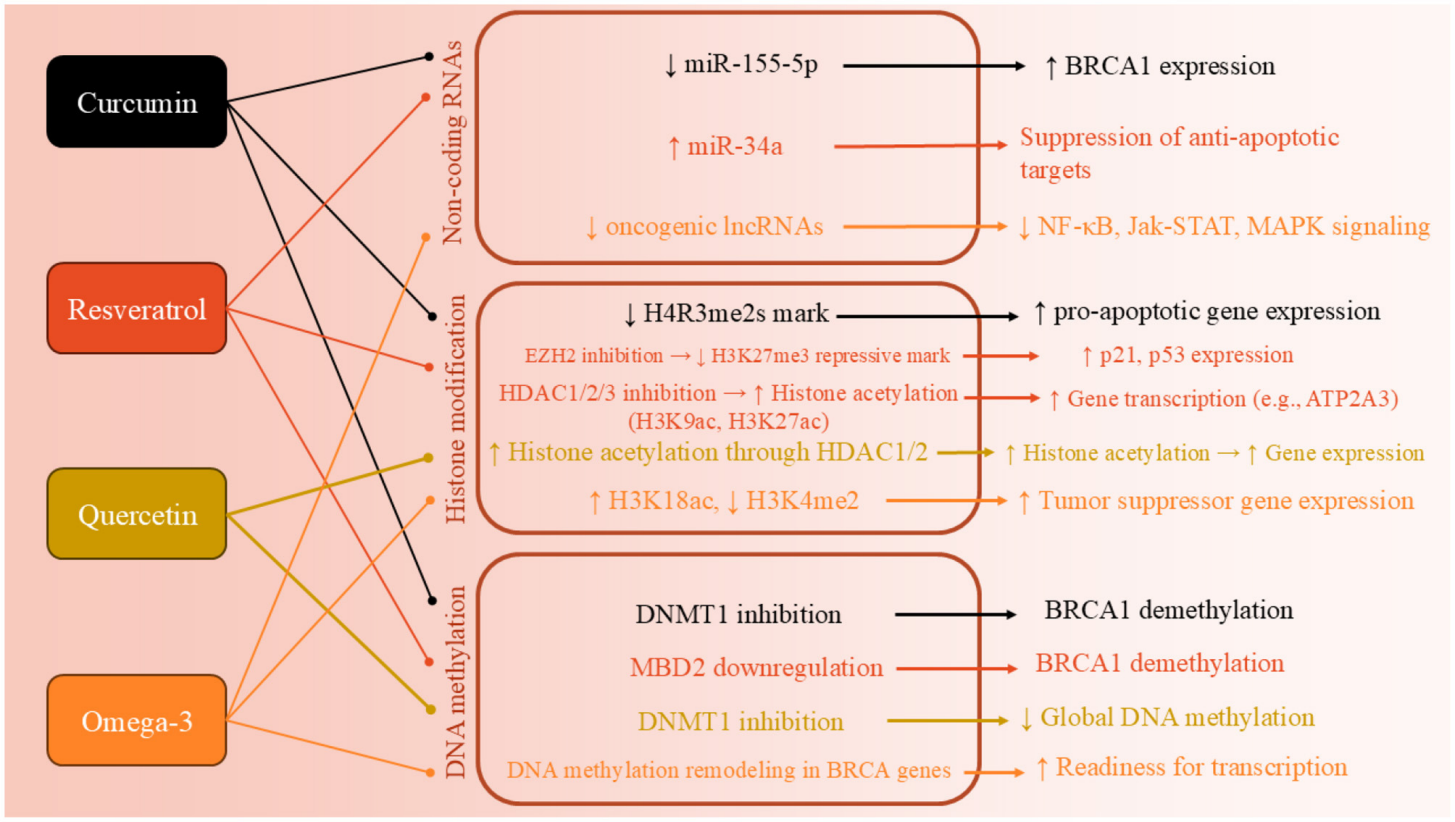
Epigenetic mechanisms by which functional foods modulate breast cancer pathways. This figure illustrates how selected functional foods—including curcumin, resveratrol, quercetin, omega-3 fatty acids, and vitamins—exert anticancer effects through specific epigenetic mechanisms. These include inhibition of DNA methyltransferases (DNMTs), suppression of histone-modifying enzymes (e.g., EZH2, HDACs, PRMT5), modulation of non-coding RNAs (e.g., miRNAs, lncRNAs), and histone acetylation enhancement. The downstream effects lead to reactivation of tumor suppressor genes (e.g., BRCA1, p21, p53), suppression of oncogenes (e.g., BCL-2, VEGF), promotion of apoptosis, reduced proliferation, and improved therapeutic response in breast cancer. These mechanisms highlight the potential of dietary bioactives in epigenetic reprogramming and precision nutrition-based cancer care.

Functional foods have emerged as a valuable complementary approach in BC prevention and management due to their capacity to modulate biological processes involved in tumor development and progression. Bioactive compounds found in foods such as curcumin, resveratrol, quercetin, omega-3 fatty acids, vitamins, and probiotics can influence gene expression through epigenetic mechanisms, including DNA methylation, histone modification, and non-coding RNA regulation (also shown in Figure 4). These compounds also exert anti-inflammatory, antioxidant, pro-apoptotic, and anti-proliferative effects, contributing to reduced tumor growth and improved response to conventional therapies. Beyond molecular modulation, functional foods support immune system function, mitochondrial efficiency, and metabolic reprogramming, factors that are critical in both tumor suppression and recovery from treatment-induced damage. Clinical and preclinical evidence suggests that regular intake of these foods can enhance chemotherapy tolerance, reduce oxidative damage, and alleviate treatment-related side effects, such as fatigue and gastrointestinal dysfunction. Exercise, likewise, plays a crucial role in modifying BC risk and outcomes. Aerobic and resistance training have been shown to lower circulating estrogen levels, improve insulin sensitivity, boost immune cell activity, and regulate key metabolic and hormonal pathways. Exercise also positively affects mental health, physical function, and quality of life in BC patients and survivors. Importantly, the combination of functional foods and exercise interventions appears to have a synergistic effect, offering more pronounced benefits than either strategy alone. Together, they enhance epigenetic remodeling, reduce systemic inflammation, improve body composition, and bolster the immune response. For instance, studies combining omega-3 supplementation with resistance training have demonstrated improvements in lean body mass and reductions in fatigue, while curcumin combined with physical activity may enhance antioxidant defenses and modulate key apoptotic genes. Emerging evidence also suggests that this combination may be effective in reversing treatment resistance and minimizing recurrence risks.

While findings are promising, challenges remain. These include variability in dosing, bioavailability of certain compounds, individual genetic and metabolic differences, and the need for standardized intervention protocols. Future research should prioritize well-designed, large-scale clinical trials to establish personalized, evidence-based guidelines for integrating functional nutrition and exercise into BC care. In conclusion, leveraging the synergistic potential of functional foods and exercise offers a promising, non-pharmacological strategy to enhance therapeutic outcomes, reduce recurrence, and improve survivorship in BC patients. A precision-based approach incorporating diet and physical activity into conventional treatment paradigms may redefine supportive care and long-term disease management in oncology.
